# Proteomics Analysis Reveals the Implications of Cytoskeleton and Mitochondria in the Response of the Rat Brain to Starvation

**DOI:** 10.3390/nu11020219

**Published:** 2019-01-22

**Authors:** Beatriz Cuevas-Fernández, Carlos Fuentes-Almagro, Juan Peragón

**Affiliations:** 1 Biochemistry and Molecular Biology Section, Department of Experimental Biology, University of Jaén, Campus Las Lagunillas, 23071 Jaén, Spain; b02cufeb@gmail.com; 2 Proteomics Unit, Central Service of Support to Research, University of Córdoba (SCAI), 14014 Córdoba, Spain; b72fualc@uco.es

**Keywords:** brain, proteomics, starvation, 2-D, mitochondria, cytoskeleton

## Abstract

Long-term starvation provokes a metabolic response in the brain to adapt to the lack of nutrient intake and to maintain the physiology of this organ. Here, we study the changes in the global proteomic profile of the rat brain after a seven-day period of food deprivation, to further our understanding of the biochemical and cellular mechanisms underlying the situations without food. We have used two-dimensional electrophoresis followed by mass spectrometry (2D-MS) in order to identify proteins differentially expressed during prolonged food deprivation. After the comparison of the protein profiles, 22 brain proteins were found with altered expression. Analysis by peptide mass fingerprinting and MS/MS (matrix-assisted laser desorption-ionization-time of flight mass spectrometer, MALDI-TOF/TOF) enabled the identification of 14 proteins differentially expressed that were divided into 3 categories: (1) energy catabolism and mitochondrial proteins; (2) chaperone proteins; and (3) cytoskeleton, exocytosis, and calcium. Changes in the expression of six proteins, identified by the 2D-MS proteomics procedure, were corroborated by a nanoliquid chromatography-mass spectrometry proteomics procedure (nLC-MS). Our results show that long-term starvation compromises essential functions of the brain related with energetic metabolism, synapsis, and the transmission of nervous impulse.

## 1. Introduction

Long-term starvation, implying the lack of nutrient intake, triggers a complex physiological and biochemical reaction that involves an adaptive response of all organs and tissues, including the integrative systems. This adaptation involves responses of the central and peripheral nervous system together with the response of the endocrine system [1]. Different studies have shown that the aim of this global adaptive response is the conservation of energy or fuels to preserve the availability of cellular ATP levels for the functions of the different tissues. The body is forced to minimize oxidative damage and maintain a metabolic balance to survive the starvation period [1,2,3]. The information reported to date suggests the adaptive response attempts to protect the brain from the effect of nutrient deficiency and; thereby, prevent irreversible brain damage.

During starvation, the expression of various neuronal genes in the hypothalamus is regulated to change their hormonal and metabolic behavior, to assume a state of positive energy equilibrium [4]. Several studies have demonstrated that the main energy fuel consumed by the brain is glucose. During starvation, this substrate must be derived from carbohydrate reserves, from *de novo* gluconeogenesis from amino acids, glycerol, or lactate [5]. Today it is known that, during starvation, the function of the brain can be maintained also by the oxidation of ketone bodies. Particularly in the brain, ketones are a source of carbon for glutamate (anaplerosis), thus help to balance glutamate homeostasis through the stabilization of energy metabolism in astrocytes after recovery from a hypoxic/ischemic event [6].

Moreover, it has been demonstrated that, after a reduction in food intake (dietary restriction) or nutrient-sensing pathways, a longer life span and protection against age-related diseases and neurodegeneration result in rodents [7]. A similar effect is observed during starvation [8]. Thus, dietary restriction, starvation, and reduced activity of nutrient-sensing pathways may slow ageing by different mechanisms, including effects on the insulin/insulin-like growth factors (IGF) signaling pathway [7]. Thus, dietary restriction and starvation can reportedly offer an advantage for the organisms and their nervous system. 

Proteomics has developed to become a useful tool in nutritional and clinical research. Its aim is the complete and quantitative description of overall protein expression and its changes, which allow their correlation with the underlying biological processes. Different aspects, such as the type of proteins expressed, their abundance, and the state of modification of these proteins, depends on the physiological and pathophysiological state of the cell or tissue. Therefore, the proteome reflects the state of the cell, which depends on the external and internal conditions. Caloric restriction and starvation are two nutritional states that have been investigated, generally in relation to aging and diseases such as obesity and diabetes [9,10,11]. 

An exhaustive search in the scientific literature revealed no studies so far on global proteomics of the brain after a long-term starvation. The brain, a fundamental organ for the survival of the organism, has special energy requirements. The aim of this study is to identify the main proteins differentially expressed in the brain during a long-term starvation by means of 2-D and MS analysis (MALDI-TOF/TOF). The results were validated by performing another proteomic procedure based on nanoliquid chromatography and MS analysis (nLC-MS). The results might provide insights into the cell functions that are affected or that start being affected in the brain after a period of prolonged starvation.

## 2. Materials and Methods

### 2.1. Chemicals and Drugs

Specific reagents used for making buffers and other solutions came from Sigma-Aldrich Chemical Co. (St. Louis, MO, USA) and Fluka (Buchs SG, Switzerland). Chemicals and materials used for 2-D analysis were supplied by BioRad Laboratories (Hercules, CA, USA) and GE Healthcare (Uppsala, Sweden). 

### 2.2. Animals and Experimental Design

The experiment, conducted in the Animal Production and Experimentation Center of the University of Jaén (Spain), was reviewed and approved by the Ethics Committee of the University of Jaén as well as the Ethics Committee of the Junta de Andalucía (Spain). All procedures were performed in accordance with national and international guidelines for animal experimentation. 

For this experiment, 18 male Wistar rats, with an average weight of 390.81 ± 6.23 g, were divided into 2 groups of 9 rats, each with 3 rats per cage (3 cages per group). The rats were maintained under controlled lighting conditions (12 h light/12 h darkness cycle) and temperature (22 °C), and had free access to water and a standard diet (Harlan, Ref. T.2014.12) for 7 days of adaptation. The composition of the diet was: crude protein 14.3%, fat 4%, digestible carbohydrates 48%, fiber 22.1%, and energy 12.1 kJ/g. Then, for one of the groups, called “starvation”, the meal was removed for 7 days. The other group, called “control”, was maintained with free access to the standard diet for 7 days. After rats were killed by cervical dislocation, the brain was immediately removed, put on ice, and then washed with saline solution (NaCl 0.9%, *p*/*v*), weighed, and finally frozen in liquid nitrogen until the preparation of the samples.

### 2.3. Protein Extraction for 2-D

For protein extraction, the brains of the 3 rats from the same cage were pooled. Next, 0.2 g of brain tissue from the control and starved rats were homogenized with 2 mL of buffer containing 8 M urea, 2 M thiourea, 4% 3-[(3-cholamidopropyl)dimethylammonio]-1-propanesulfonate hydrate (CHAPS), 2% isoelectric focusing gel (IPG) buffer, 20 mM dithiothreitol (DTT), 100 mM HCl-Tris, and 0.75 mM phenylmethylsulfonyl fluoride (PMSF) (pH 8). The homogenate was shaken at 4 °C gently for 1 h. During this time the samples were moderately shaken in a vortex every 15 minutes. The homogenates were centrifuged at 10,000× *g* for 15 min at 4 °C. The supernatants were cleaned using Ready 2-D CleanUp kit (BioRad Laboratories, Hercules, CA, USA) and the resulting samples were used for 2-D and nLC-MS. The protein concentration was measured by CB-X^TM^ Protein Assay (G-Biosciences, St Louis, MO, USA). Three replicates of the homogenates were prepared per experimental group, each being made up of 3 different rats.

### 2.4. 2-D

Protein extracts (300 μg) were incubated for 30 minutes in 450 μL of rehydration buffer containing 7 M urea, 2 M thiourea, 4% 3-[(3-cholamidopropyl)-dimethylammonium]-1-propane sulphonate (CHAPS), 30 mM DTT, 0.5% Pharmalyte 5–8, and bromophenol blue traces. The samples were loaded onto 17 cm (pH 4–7) Immobiline DryStrips (GE Healthcare Bio-Sciences AB, Uppsala, Sweden). After 2 h passive and 10 h active (50 V) rehydration in a Protean IEF Cell (BioRad Laboratories, Hercules, CA, USA) at 20 °C and 50 μA/strip, the voltage was raised to 1000 and 4000 V in a 120-minutes gradient, and to 8000 V in 30 min. The sample was maintained at 8000 to 50,000 V h for around 6 h. After isoelectric focusing, the strips were soaked for 15 min in an equilibration buffer containing 50 mM Tris-HCl (pH 8.8), 6 M urea, 30% glycerol, 2% sodium dodecyl sulfate (SDS), bromophenol blue traces, and 20 mM DTT. The strips were then drained and soaked again for a further 15 min in the same mix plus 25 mM iodoacetamide. SDS-PAGE was undertaken in 12% gels using the Protean Plus Dodeca cell (BioRad Laboratories, Hercules, CA, USA) at 20°C and 2.5 watts/gel for 10 min, and 10 watts/gel until separation was complete. The gels were stained with Sypro Ruby^R^ protein stain (BioRad Laboratories, Hercules, CA, USA) compatible with MS analysis.

### 2.5. Quantitative Analysis of Gel Images and Statistical Analysis

Gel images of three replicates per sample were made with a BioRad FX Pro Plus densitometer (BioRad Laboratories, Hercules, CA, USA). Spot volumes, normalized by the total volume of all the validly matched spots in a set of gels, were quantified using the PDQuest Advanced software (BioRad Laboratories, Hercules, CA, USA). Only spots showing at least a two-fold over-/under-expression ratio, compared to any other sampling site, were taken into account. One-way analysis of variance (ANOVA) followed by Student’s *t*-test were then used to choose the spots that showed altered expression patterns between the two experimental groups.

### 2.6. Protein Digestion and MS Analysis

Differentially expressed spots were automatically excised in an Investigator ProPic (Genomic Solutions, Cambridgeshire, UK). The gel pieces were digested with trypsin using a ProPrep II Automated Protein Digestion instrument (Genomic Solutions, Investigator Digilab, Cambridgeshire, UK) according to the following process: two 30-min destaining steps with 50% acetonitrile (ACN)/100 mM ammonium bicarbonate, two 15-min washes with 25 mM ammonium bicarbonate/50% ACN, dehydration for 5-min with 100% ACN, followed by drying. The sample was subsequently hydrated with 10 μL of 12.5 ng μL^−1^ trypsin in ammonium bicarbonate for 45 min at 4 °C before being finally digested in a microwave for 2 times of 5 min. Digestion was stopped by adding 1 μL of 10% trifluoroacetic acid (TFA). The resulting peptides were purified in a Pro MS device (Genomic Solutions, Cambridgeshire, UK), with a C18 microcolumn (ZipTip, Millipore, Billerica, MA, USA), and eluted with a matrix solution of 5 mg mL^−1^ α-ciane-4-hydroxycinnamic acid dissolved in 70% ACN/0.1% TFA. One μL aliquots of the eluted samples were directly spotted onto MALDI plates.

Mass analysis (MS) of the peptides in each sample was undertaken with a MALDI-TOF/TOF mass spectrometer (4800 Proteomics Analyzer, Applied Biosystems Carlsbad, CA, USA), in the *m*/*z* range of 800 to 4000 *m*/*z* and with an accelerating voltage of 20 kV. Spectra were internally calibrated with peptides from trypsin autolysis (M+H^+^ = 842.509, M+H^+^ = 2211.104). The most abundant peptide ions were then subjected to fragmentation analysis (MS/MS) to provide information for use in determining the peptide sequence.

### 2.7. Database Searching

Proteins were identified by peptide mass fingerprinting, which was confirmed by MS/MS analysis. The Mascot 2.0 search engine (Matrix Science Ltd., London, UK) was used for protein identification, running on the GPS Explorer^TM^ software v3.5 (Applied Biosystems, Carlsbad, CA, USA) to search in the National Center for Biotechnology Information (NCBI) protein database (updated monthly).

The search setting allowed one missed cleavage with the selected trypsin enzyme, a MS/MS fragment tolerance of 0.2 Da, a precursor mass tolerance of 100 ppm, and cysteine carbamidomethylation and methionine oxidation as possible modifications. Proteins showing statistically significant (*p* < 0.05) changes in their expression were assigned positive identification after taking molecular-mass (*Mr*) and isoelectric-point (*pI*) values into consideration.

To make a classification of the protein–protein interactions of the differentially expressed proteins and to determine the interactions, the network STRING v10.0 (Protein–Protein Interaction Networks: http://string-db.org/cgi/input.pl) was used. Pre-established values of the software were used to obtain the proteins associations. 

### 2.8. nLC-MS Proteomics Procedure

#### 2.8.1. Sample Preparation

Protein extracts were cleaned in 1D SDS-PAGE at 10% of polyacrylamide. Samples were loaded in stacking gel and 100 V was applied until the electrophoresis front reached the resolving gel. After the protein extracts were separated by 1 cm in resolving gel, the electrophoresis was finished and the gel was stained with Coomassie Blue. Protein bands were excised, diced, and kept in water until digestion.

#### 2.8.2. Protein Digestion

Briefly, protein bands were firstly destained in 200 mM ammonium bicarbonate (AB)/50% acetonitrile for 15 min and 5 min in 100% acetonitrile. Protein was reduced by addition of 20 mM dithiothreitol in 25 mM AB and incubated for 20 min at 55 °C. The mixture was cooled to room temperature, followed by alkylation of free thiols by addition of 40 mM iodoacetamide in 25 mM BA in the dark for 20 min. Afterwards, protein bands were washed twice in 25 mM AB. Proteolytic digestion was performed by adding trypsin (Promega, Madison, WI, USA), 12.5 ng/ul of enzyme in 25 mM AB, and incubating at 37 °C overnight. Protein digestion was stopped by adding trifluoroacetic acid at 1% final concentration, and the samples were dried in SpeedVac.

#### 2.8.3. nLC-MS2 Analysis

Briefly, four technical replicates per sample were performed to validate changes detected in 2D differential expression. Nano-LC was performed in Dionex Ultimate 3000 nano UPLC (Thermo Scientific) with a C18 75 μm x 50 cm Acclaim Pepmam column (Thermo Scientific). Previously, the peptide mix was loaded in a 300 µm x 5 mm Acclaim Pepmap precolumn (Thermo Scientific) in 2% acetonitrile/0.05% TFA for 5 min at 5 µl/min. Peptide separation was performed at 40 °C for all runs in. Mobile phase buffer A was composed of water plus 0.1% formic acid. Mobile phase B was composed of 80% acetonitrile plus 0.1% formic acid. Samples were separated at 300 nL/min. The mobile phase B increased to 4% to 45% B for 60 min, and 45% to 90% B for 1 min, followed by a 5-min wash at 90% B, and a 15-min re-equilibration at 4% B. The total time of chromatography was 85 min.

After elution the peptide cations were converted to gas-phase ions by nano electrospray ionization, and analyzed on a Thermo Orbitrap Fusion (Q-OT-qIT, Thermo Scientific). The mass spectrometer was operated in the positive mode. Survey scans of peptide precursors from 400 to 1500 *m*/*z* were performed at 120K resolution (at 200 *m*/*z*) with a 5 × 10^5^ ion count target. Tandem MS was performed by isolation at 1 Th with the quadrupole, collision-induced dissociation CID fragmentation with normalized collision energy of 35, and rapid scan MS analysis in the ion trap. The automatic gain control (AGC) ion count target was set to 102 and the max injection time was 75 ms. Only the precursors with charge state 2–5 were sampled for a second in-tandem mass analysis MS2. The Dynamic Exclusion duration was set to 15 s with a 10-ppm tolerance around the selected precursor and its isotopes. Monoisotopic precursor selection was turned on. The instrument was run in Top Speed Mode with 3-s cycles, meaning that the instrument would continuously perform MS2 events until the list of nonexcluded precursors diminished to zero or 3 s, whichever was shorter. 

#### 2.8.4. Data Analysis

The raw data were processed using Proteome Discoverer (version 2.1.0.81, Thermo Scientific). MS2 spectra were searched with SEQUEST HT engine against a database of Uniprot_Musmusculus_Dic2016 (www.uniprot.org). Peptides were generated from a tryptic digestion with up to one missed cleavage, carbamidomethylation of cysteines as fixed modifications, and oxidation of methionines as variable modifications. Precursor mass tolerance was 10 ppm and product ions were searched for at 0 Da tolerances. Peptide spectral matches (PSM) were validated using percolator based on *q*-values at a 1% false discovery rate (FDR). With Proteome Discoverer, peptide identifications were grouped into proteins according to the law of parsimony and filtered to 1% FDR.

#### 2.8.5. Quantitative First-Mass MS1 Data Analysis in Skyline Software

First-mass analysis (MS1) chromatogram-based quantitation was conducted in Skyline 3.6.0.10162 (24), an open-source software project (http://proteome.gs.washington.edu/software/skyline), as recently described in detail for MS1 filtering [12]. First, comprehensive spectral libraries were generated in Skyline from database searches from Proteome Discoverer 2.1, of the raw data files, prior to MS1 filtering. Second, all raw files acquired in data dependent acquisition (DDA) mode were directly imported into Skyline, and MS1 precursor ions were extracted for all peptides present in the MS/MS spectral libraries. Quantitative MS1 analysis was based on extracted ion chromatograms (XICs) and was made for the top three resulting precursor ion peak areas (e.g., M, M+1, and M+2). Final quantitative comparisons were typically based on only the highest ranked precursor ion. After data import, graphical displays of chromatographic traces (extracted ion chromatograms) were manually inspected for proper peak picking of MS1 filtered peptides. In some cases, the peak integration was adjusted manually in the chromatographic window. Each peptide area of each protein was added together in order to calculate protein abundances in all samples. Fold changes and statistical analysis were calculated with MSstats, an R package involved in Skyline software [13]. The statistical significance of each protein ratio was indicated by its adjusted *p*-value (<0.1).

## 3. Results

**Figure 1:**
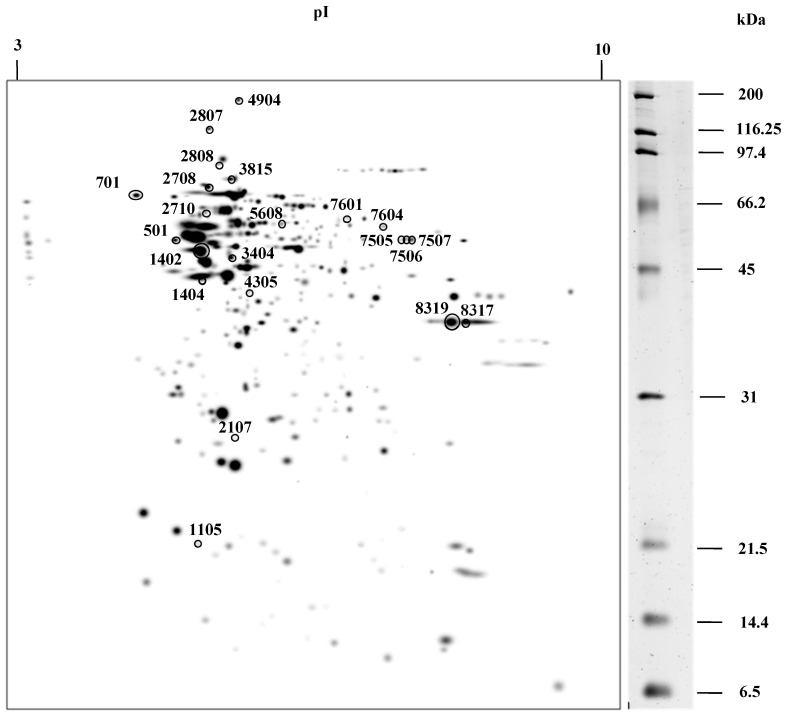
Master gel obtained from 2-D of the rat brain proteins under control and starvation conditions. The 22 spots that show a differential expression are circled. These were excised and digested with trypsin. The peptides obtained were analyzed by a matrix-assisted laser desorption-ionization-time of flight mass spectrometer MALDI-TOF/TOF.

Brain protein extracts from control and starved rats were analyzed by 2-D. For each of the situations, three gels were made, for a total of six gels. Figure 1 shows the master gel in which the spots detected in the six gels were considered. Approximately 346 spots were detected in the master gel and the number of spots detected on other gels ranged from 300 to 308. The proteins detected had a molecular weight between 6.5 and 200 kDa, while the isoelectric point (pI) of the protein ranged from 3.2 to 9.4.

Of all the detected proteins, those that were differentially expressed in the two conditions were selected. For the choice of proteins, several criteria were considered: There was a two-fold difference in protein abundance in one condition vs. the other, and this change was repeated in the three replicates analyzed from each group of rats. The quantitative analysis of the gels resulted in 22 spots with significant differential expression, as shown in Figure 1.

The results for the MS and MS/MS analyses, after a combined search in the databases, are presented in Table 1. Protein identification was based on the homology of three peptides by searching for these in the databases, limiting the search to mammals. Most of the homologies found had a good molecular weight search (MOWSE) score and high sequence (homology) coverage. The scoring criterion is given by the statistical significance that the MASCOT search engine calculates for each identification. A total of 14 spots were identified from protein or cDNA sequences described in mammalian species, such as *Rattus norvegicus*, *Mus musculus*, *Oryctolagus cuniculus*, *Monodelphis domestica*, and *Heterocephalus glaber*. Good homology results were found for 64% of the 22 spots analyzed, while the peptide footprint of the remaining eight spots did not meet the criteria established for positive identification.

The spots identified corresponded to 13 different proteins. In the case of glyceraldehyde-3-phosphate dehydrogenase (GAPDH), two spots were identified with two different variants of the same protein. The identified variants were located in the horizontal direction of the gel and; therefore, their presence could be due to the charge change caused by the post-translational alterations.

The functions of these proteins, taken from the UniProt database (http://www.uniprot.org/), are shown in Table 2. 

**Figure 2:**
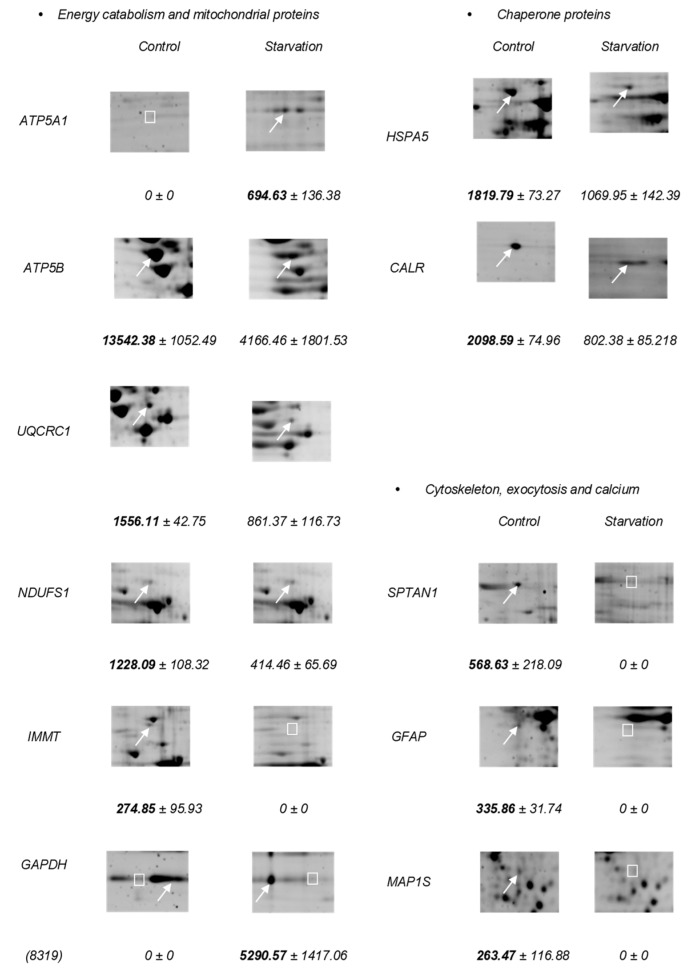
Enlarged images of 2-D gels that show the differential expression of 14 proteins identified in the rat brain. The arrows point to the spots and the squares indicate the absence of spots. Numbers in bold indicate that this spot is present in greater quantity than the other condition.

The proteins identified were grouped into three categories depending on their function or participation in metabolic pathways (Figure 2): 

(1) Energy catabolism and mitochondrial proteins, including the α subunit of ATP synthase (ATP5A1), the β subunit of ATP synthase (ATP5B), subunit 1 of the cytochrome b-c1 complex (UQCRC1), the subunit of 75 kDa of NADH-ubiquinone oxidoreductase (NDUFS1), MIC60 subunit of the mitochondrial contact site and cristae organizing system (MICOS) (internal mitochondrial membrane protein) (IMMT), and glyceraldehyde-3-phosphate dehydrogenase (GAPDH) complex;

(2) Chaperone proteins, including 78 kDa glucose-regulated protein (HSPA5) and calreticulin (CALR);

(3) Cytoskeleton, exocytosis, and calcium, such as the α-chain of non-erythrocytic spectrine 1 (SPTAN1), glial fibrillary acidic protein (GFAP), 1S microtubule-associated protein (MAP1S), Secernin-1 (SCRN1), and melanoma-associated antigen (MAGEA11).

Figure 2 shows the proteins identified and grouped into different categories with differential expression. Except for some cases, there was a general fall in expression levels of brain proteins after the starvation period. In the Group 1, the long-term food deprivation caused a decrease of more than two-fold in the expression of ATP5B, UQCRC1, and NDUFS1. On the other hand, ATP5A1 changed from not expressing itself in the control situation to an expression of 694.63 units of optical density (O.D) after the starvation period. In the case of IMMT, food deprivation caused the complete disappearance of protein expression, going from 274.85 to 0. Two isoforms of GAPDH were also shown; the isoform 8319 went from having no expression in the control situation to registering an increase to 5290.57 in the starvation situation, while the isoform 8317 went from having an expression of 3007.11 in the control situation to having no expression after the starvation period. Here we see a clear substitution of isoform 8317 to 8319 of GAPDH, caused by the experimental situation. Within the Group 2 of proteins, HSPA5 decreased its expression during starvation, and CALR also reduced its expression by more than double after starvation. The most striking changes occurred in the proteins of Group 3, where all the proteins were not expressed in the starvation situation. 

**Figure 3:**
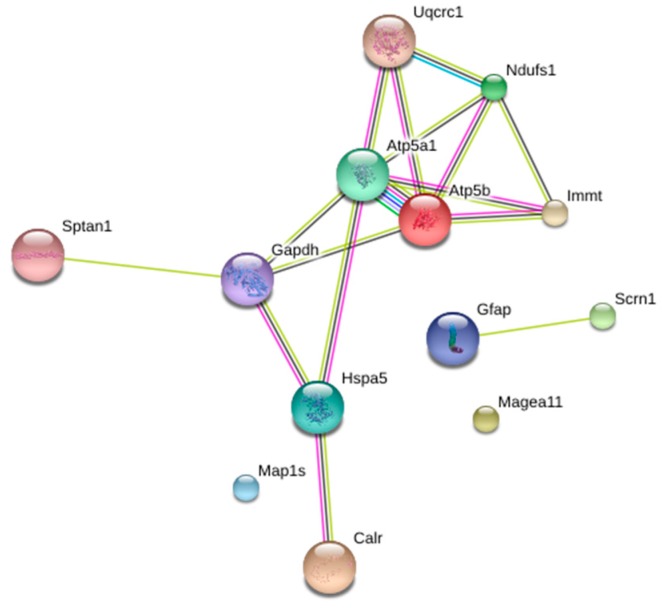
Rat-brain protein–protein interaction network differentially expressed after seven days of starvation. These data were obtained using STRING program, adjusting the classification to the first five categories.

Figure 3 shows a classification according to the protein–protein interactions of brain proteins differentially expressed after the starvation period. This is the classification provided by the STRING software, considering only the first 5 categories. Within the clusters: biological processes, molecular functions, cellular components, and KEGG (Kyoto Encyclopedia of Genes and Genomes) routes, we found proteins that were included in several groups simultaneously. MAP1S is also included in this classification, although this protein does not interact with any of the other proteins. Of special interest was the classification of “KEGG routes”, where the proteins ATP5A1, ATP5B, NDUFS1, and UQCRC1 participate in both oxidative phosphorylation and diseases such as Parkinson’s, Alzheimer’s, and Huntington’s.

**Figure 4:**
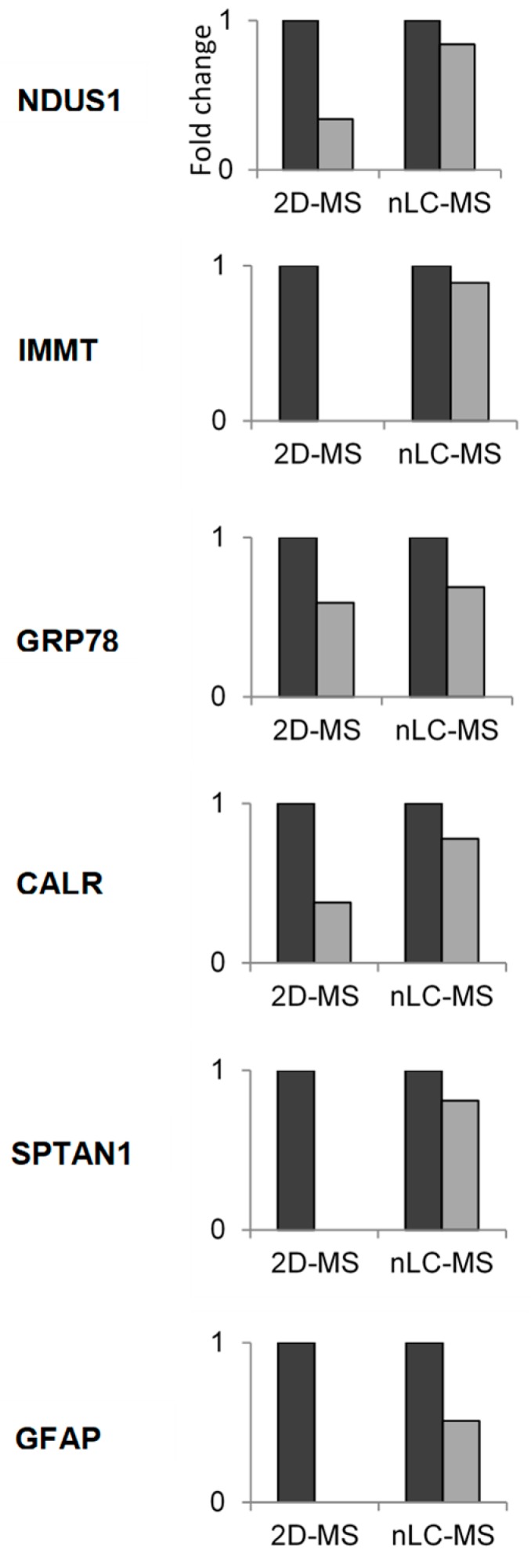
Fold change abundance determined by nanoliquid chromatography-mass spectrometry proteomics procedure nLC-MS of some proteins differentially expressed by two-dimensional electrophoresis followed by mass spectrometry (2D-MS) in response to starvation in the rat. Results are expressed as mean ± S.E.M. The protein-expression level in the starvation (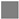) is expressed against control value (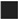) that is considered with the value of 1. In all cases the differences between control and starvation were significant.

The 2D-MS data were validated by quantifying the fold change detected in selected proteins using a nLC-MS proteomics procedure. As shown in Figure 4, the protein-expression level from NDUS1, IMMT, GRP78, CALR, SPTAN1, and GFAP fell after long-term starvation. The resulting protein-expression patterns were consistent with those found by 2D-MS analysis, confirming the results reached with this procedure. 

## 4. Discussion

In this work, we studied the differential expression of rat brain proteins after a long-term starvation. These proteins were separated by 2-D electrophoresis and identified by MALDI-TOF/TOF. With the help of specialized software, proteins differentially identified according to their cellular function were classified and grouped according to the networks of interactions between them. With the results, we gain overall knowledge of the main changes in brain protein expression that occur in response to starvation.

The metabolic adaptation that the brain undergoes during starvation leads to changes in protein expression. The results of this study show the existence of 14 proteins differentially expressed and classified into three groups: (1) energy catabolism and mitochondrial proteins, (2) chaperone proteins, and (3) cytoskeleton, exocytosis, and calcium.

The lack of the intake of nutrients provokes mitochondrial dysfunction, involving several proteins that show diminished expression: the ATP synthase α subunit (ATP5A1), the ATP synthase β subunit (ATP5B), the cytochrome b-c1 subunit 1 (UQCRC1), the 75-kDa subunit of NADH-ubiquinone oxidoreductase (NDUFS1), and internal mitochondrial membrane protein (IMMT). ATP5A1, ATP5B, UQCRC1, and NDUFS1 form part of the mitochondrial respiratory chain. ATP5A1 and ATP5B are part of the ATP synthase, which is responsible for the ATP production [14,15]; UQCRC1 is a component of the ubiquinole-cytochrome c reductase complex (Complex III) [16], while NDUFS1 is the core subunit of NADH dehydrogenase (Complex I) [17]. The protein IMMT maintains the internal architecture of the membrane and mitochondrial ridges, in addition to forming sites of contact with the external mitochondrial membrane, so that the function of this protein is to maintain the integrity of the mitochondria [18,19]. Energy catabolism in the brain is also affected, as demonstrated by the decreasing expression of glyceraldehyde-3-phosphate dehydrogenase (GAPDH) under starvation conditions. GAPDH is a key enzyme in glycolysis that catalyzes the conversion of glyceraldehyde-3-phosphate to 1,3-bisphosphoglycerate [20].

Some chaperones such as the 78kDa glucose-regulated protein (HSPA5) and calreticulin (CALR) are also affected by the starvation period, by decreasing expression. HSPA5 is a chaperone that facilitates the assembly of multimeric protein complexes in the endoplasmic reticulum [21], and CALR is a calcium-binding chaperone that promotes protein folding, oligomeric assembly, and the quality control of proteins in the endoplasmic reticulum by the calreticulin/calnexin cycle [22,23]. CALR and HSPA5 chaperones are related to the activation of the stress response of the endoplasmic reticulum. At the beginning of the response, chaperones seek to restore homeostasis in the endoplasmic reticulum, but if they fail to achieve this balance, they attempt to trigger cell death [21,23]. 

The expression of some cytoskeleton-associated proteins also declines or disappears, such as the non-erythrocytic spectrin 1 α chain (SPTAN1), the glial fibrillary acidic protein (GFAP), and the microtubule-associated 1S protein (MAP1S). SPTAN1 interacts with calmodulin in a calcium-dependent way and may participate in the calcium-dependent movement of the cytoskeleton to the membrane [24], GFAP is a cytoskeleton-specific protein of astrocytes [25] and MAP1S modulates the function of microtubules and promotes their stability in mammalian cells [26]. Secernin-1 (SCRN1) and melanoma-associated antigen 11 (MAGEA11), as well as the above-mentioned proteins, show no levels of expression during the starvation period. SCRN1 regulates exocytosis in mast cells [27] and MAGEA11 acts as a co-regulator of the androgen receptor, stimulating its activity [28].

These expression changes, in the larger context of cellular functions, provide key information concerning events in the brain during starvation. The gradual decline observed in the expression of proteins involved in mitochondrial energy catabolism may be related to less use due to the lack of metabolic fuels, involving a lower respiratory rate and a lower need for such use. This even affects proteins involved in the maintenance of membrane structure. Some of these proteins are also involved in neurodegenerative diseases as widespread as Parkinson’s, Alzheimer’s, or Huntington’s disease, implying the alteration of oxidative phosphorylation and mitochondrial structure in these diseases. 

In specific cases, this change of expression can be explained individually. The two isoforms of GAPDH are interchanged in response to starvation. The 8317 isoform, expressed in control, is replaced by 8319, expressed during starvation. This change may be prompted due to GAPDH, which, in addition to its role in glycolysis, is a multifunctional protein involved in oxidative and nitrosative stress in the brain, being related to neurological disorders [29].

Moreover, all proteins related to the cytoskeleton diminish their expression until disappearing after starvation. SPTAN1 and MAP1S regulate the assembly in the cytoskeleton and play an important role in the neuronal synapse [23,30,31,32]. The lack of expression of these proteins shows that the synapses malfunction in the brain. The decrease in the expression of proteins involved in the cytoskeleton, exocytosis, and calcium binding implies the existence of an alteration in the vesicle transport, which is essential in transmitting neurotransmitters and transmitting nerve impulses, both inside the neuron as well as across the synapse. GFAP is an astrocyte-specific cytoskeletal protein whose expression increases during reactive astrogliosis, which occurs in response to damage to the central nervous system [25,33]. According to our results, GFAP is not expressed during starvation, indicating a fall in astrocyte production in this situation.

Long-term food deprivation causes changes at the cellular and intracellular levels. In this work, we found that proteins associated with key cellular functions are affected. Mitochondria and cytoskeleton are the main cellular constituents that showed the greatest functional changes in response to starvation. Mitochondria prove critical in several metabolic functions such as lipid metabolism and energy production. This organelle is essential for the aerobic production of ATP, β-oxidation of fatty acids, ketogenesis, and gluconeogenesis from pyruvate and Krebs cycle intermediates. Severe malnutrition can cause dysfunction in mitochondria, which in turn provokes liver disorders due to oxidative stress and hepatic steatosis [34]. Changes in mitochondrial dynamics have also been linked to neurodegenerative diseases. When mitochondria malfunction, processes such as oxidative phosphorylation and calcium regulation falter, causing problems in the neuronal synapses [35]. Moreover, different works have related starvation, caloric restriction, and perturbations in mitochondrial function with lifespan and ageing [36,26]. 

In addition, the cytoskeleton and its components are vital for transport in different biological processes, and these pathways hydrolyze ATP to provide mechanical energy. Cells use the elements of the cytoskeleton to transport small molecules, macromolecules, and organelles to the site where they will serve their biological function. This transport is fundamental in the polarization, extension, shape, and neurotransmission of the neurons. The elements of the cytoskeleton, such as myosin, kinesin, and dynein, work together to maintain the actin and microtubule filament system that ensures the appropriate transport and proper structural basis in the cells. Dysfunction of cytoskeletal proteins upsets the transport and harms cellular morphology, which is related to different maladies such as neurodegenerative diseases [35,37].

In our experiment we do not perform specific analysis to correlate the changes in protein abundance with synapses’ or motor malfunction that can be reflected in changes in movement capacity or in an abnormal behavior. Nevertheless, it is well known that food restriction induces hyperactivity in rats and other rodents [38]. In relation with a rat model of anorexia nervosa, a reduced availability of food stimulates running in a wheel, and excessive running induces self-starvation. In this vicious circle, rats actually starve and run themselves to death [38]. These changes in the behavior can be the result of a brain malfunction also induced by starvation in rat.

In some tissues or cell types, an important adaptation to starvation is autophagy that can be initiated by nutrients and amino acid deprivation, between other signals [39,40]. It can be a global or selective process that can affect specific proteins [41]. The autophagosomes generated in this process can involve cytoplasmic material and/or organelles, such as mitochondria and breakdown for re-use amino acids and other molecules as sources of energy and nutrients [42]. It is not clear what occurs in the brain in response to starvation [43], although it has been described that autophagy can protect neurons from beta amyloid-induced cytotoxicity [44]. In our results we have not found changes in proteins involved in the regulation of autophagy in response to seven-days starvation in the rat brain, implying that this food deprivation situation does not produce detectable autophagy in brain. Nevertheless, the decrease in the abundance of mitochondrial and cytoskeleton proteins can be related with a stimulation of autophagy or another protein breakdown mechanism during starvation. In conclusion, the main changes induced by a long-term starvation in the brain proteome of the rats affect energetic metabolism, chaperone proteins, and the cytoskeleton. These changes can result in the alteration of ATP production and vesicle dynamics fundamental for the maintenance of the function of this organ. 

## Figures and Tables

**Table:** 

**BIOLOGICAL PROCESSES**	
Cytoskeleton organization	CALR, GAPDH, GFAP, MAP1S, SPTAN1
Organelle organization	CALR, GAPDH, GFAP, MAP1S, NDUFS1, SPTAN1
**MOLECULAR FUNCTIONS**	
Proteins binding complex	GAPDH, GFAP, HSPA5, SPTAN1, UQCRC1
Transport of protons for ATP synthase activity, rotational mechanisms	ATP5A1, ATP5B
Binding to unfolded proteins	CALR, HSPA5
**CELLULAR COMPONENTS**	
Part of intracellular organelles	ATP5A1, ATP5B, CALR, GFAP, HSPA5, IMMT, MAP1S, NDUFS1, SCRN1, SPTAN1, UQCRC1
Protein complex of inner mitochondrial membrane	ATP5A1, ATP5B, NDUFS1, UQCRC1
Protein complex	ATP5A1, ATP5B, GAPDH, GFAP, HSPA5, MAP1S, NDUFS1, SPTAN1, UQCRC1
Inner mitochondrial membrane	ATP5A1, ATP5B, IMMT, NDUFS1, UQCRC1
Organelle membranes	ATP5A1, ATP5B, CALR, HSPA5, IMMT, NDUFS1, SCRN1,UQCRC1
**KEGG PATHWAYS**	
Oxidative phosphorylation	ATP5A1, ATP5B, NDUFS1, UQCRC1
Parkinson’s disease	ATP5A1, ATP5B, NDUFS1, UQCRC1
Alzheimer’s disease	ATP5A1, ATP5B, NDUFS1, UQCRC1
Huntington’s disease	ATP5A1, ATP5B, NDUFS1, UQCRC1
Processing and introduction of antigens	CALR, HSPA5

**Table 1:** Brain proteins identified by fingerprinting peptide and MS/MS analysis of peptides from 2-D exscinded spots using MASCOT software.

Spot ^a)^	Homology	Species	Mw (kDa)	pI	# Peptides ^b)^	Access Number	MOWSE Score ^c)^	Coverage (%) ^d)^
501	SCRN1	*Rattus norvegicus*	46.993	4.73	8	sp|Q6AY84|SCRN1	68	21
701	CALR	*Rattus norvegicus*	48.136	4.37	16	sp|P18418|CALR_	304	39
1402	ATP5B	*Oryctolagus cuniculus*	45.549	5.21	22	tr|Q0QEN9|Q0QEN	885	63
1404	GFAP	*Rattus norvegicus*	49.983	5.35	12	sp|P47819|GFAP_	199	32
2708	HSPA5	*Mus musculus*	72.492	5.01	34	tr|Q9DC41|Q9DC4	646	53
2807	SPTAN1	*Mus musculus*	98.016	5.17	26	tr|Q3URW8|Q3URW	210	32
2808	IMMT	*Rattus norvegicus*	86.689	5.62	13	tr|A0A0G2JVH4|A	75	18
3404	UQCRC1	*Rattus norvegicus*	53.499	5.57	20	sp|Q68FY0|QCR1_	327	39
3815	NDUFS1	*Rattus norvegicus*	80.330	5.65	30	sp|Q66HF1|NDUS1	386	46
4305	MAP1S	*Monodelphis domestica*	124.777	5.97	19	tr|F7BK59|F7BK5	68	12
7506	ATP5A1	*Rattus norvegicus*	59.889	9.29	11	tr|F1LP05|F1LP0	92	23
7604	MAGEA11	*Heterocephalus glaber*	55.034	5.27	13	tr|G5C258|G5C25	66	19
8317	GAPDH	*Mus musculus*	36.072	8.44	13	sp|P16858|G3P_M	174	42
8319	GAPDH	*Mus musculus*	36.072	8.44	16	sp|P16858|G3P_M	283	50

^a)^ Reference ID number of spots. ^b)^ Number of fragmented peptides with homology. ^c)^ Molecular weight search (MOWSE) score obtained with MASCOT punctuation; punctuation corresponding to *p* < 0.001 (probability > 99.9%). ^d)^ Coverage: percentage of peptide sequence homology.

**Table 2:** Name and function of the brain identified proteins.

Abbreviation	Complete name	Functions
SCRN1	Secernine-1	Regulates exocytosis in mastocytes
CALR	Calreticuline	Calcium binding chaperone that promotes folding, oligomeric assembling and quality control in the endoplasmic reticulum by the calreticuline/calnexine cycle
ATP5B	β subunit of ATP synthase, mitochondrial precursor	ATP synthase located in the mitochondrial membrane that produce ATP from ADP
GFAP	Glial fibrillar acid protein	Specific cell target that, during development of central nervous system, distinguished astrocytes from other glia cells
HSPA5	Heat-shock protein family A (Hsp70) member 5	Facilitates the assembly of multimeric protein complex in the endoplasmic reticulum
SPTAN1	Chain α of non-erythrocyte 1 spectrine	Interacts with calmodulin in a calcium-dependent manner and could participate in the calcium-dependent movement of cytoskeleton to membrane
IMMT	Mic60 subunit of mitochondrial contact site and cristae organizing system MICOS complex, protein of the inner mitochondrial membrane	Maintenance of architecture of the inner mitochondrial membrane and formation of contact sites with external membrane
UQCRC1	Subunit 1 of mitochondrial cytochrome b-c1	Component of the ubiquinole-cytochrome c reductase
NDUFS1	75kDa subunit of NADH-mitochondrial ubiquinone oxydoreductase	Core subunit of NADH dehydrogenase
MAP1S	Protein 1S associated to microtubule	Participate in the aggregation of mitochondria from cell death and the genomic breakdown
ATP5A1	ATP synthase α subunit, mitochondrial precursor	ATP synthase from mitochondrial membrane
MAGEA11	Antigen 11 associated to melanome	Co-regulator of androgen receptor that increases its activity. Involved in calcium homeostasis in endoplasmic reticulum
GAPDH	Glyceraldehyde-3-phosphate dehydrogenase	Key enzyme of glycolysis

## Abbreviations

2-D	two-dimensional gel electrophoresis
MS	mass spectrometry
MALDI-TOF/TO	Matrix-assisted laser desorption-ionization time of flight/time of flight
nLC-MS	nanoliquid chromatography and MS analysis
GAPDH	glyceraldehyde-3-phosphate dehydrogenase
ATP5A1	α subunit of ATP synthase
ATP5B	β subunit of ATP synthase
UQCRC1	subunit 1 of the cytochrome b-c1 complex
NDUFS1	subunit of 75 kDa of NADH-ubiquinone oxidoreductase
IMMT	MIC60 subunit of the MICOS (internal mitochondrial membrane protein)
HSPA5	including 78 kDa glucose-regulated protein
CALR	calreticulin
SPTAN1	α-chain of non-erythrocytic spectine 1
GFAP	glial fibrillary acidic protein
MAP1S	1S microtubule-associated protein
SCRN1	Secernin-1
MAGEA11	melanoma-associated antigen
